# The epifascial cap: A typical imaging sign for subcutaneous granuloma annulare in children

**DOI:** 10.3389/fped.2023.1069428

**Published:** 2023-03-21

**Authors:** Besiana P. Beqo, Sebastian Tschauner, Paolo Gasparella, Iva Brcic, Emir Q. Haxhija

**Affiliations:** ^1^ Department of Paediatric and Adolescent Surgery, Medical University of Graz, Graz, Austria; ^2^ Department of Radiology, Division of Paediatric Radiology, Medical University of Graz, Graz, Austria; ^3^ Institute of Pathology, Medical University of Graz, Graz, Austria

**Keywords:** subcutaneous lesion, lumps and bumps, treatment, benign lesion, self limiting disease, children, granuloma annulare, lumps and bumps, treatment

## Abstract

**Objectives:**

Subcutaneous granuloma annulare (SGA) is a rare, self-limiting granulomatous disease in children, commonly diagnosed by histopathology following biopsy or surgical excision. This study aimed to identify imaging clues for SGA that could expedite accurate diagnosis and avoid the need for biopsy in children.

**Methods:**

We retrospectively analyzed complete hospital records of all children diagnosed with SGA at our institution from January 2001 to December 2020. Detailed disease history, imaging findings, management, and outcome were evaluated.

**Results:**

We identified 28 patients (20 girls) at a median age of 3.75 (range 1–12.5 years). Ten patients presented with multiple lesions. Most lesions were located on the lower extremities (*n* = 26/41). Ultrasound examinations were performed on all patients, and 12 (43%) patients also received an MRI. Surgical intervention was conducted in 18 (64%) patients either by incisional biopsy (*n* = 6) or total excision of the lump (*n* = 12). In all patients who did not undergo surgery, SGA resolved spontaneously. A careful review of the MRIs led to the discovery of a characteristic imaging shape of SGA lesions: the epifascial cap with a typical broad circular base laying on the fascia, extending towards the subdermal/dermal tissue. This distinctive shape was evident in every patient in our cohort.

**Conclusions:**

The “Epifascial Cap Sign” is a specific imaging sign for SGA, which to the best of our knowledge, helps distinguish this disease from other subcutaneous lesions. Recognition of this novel diagnostic sign combined with the historical and physical findings should enable clinicians to establish SGA diagnosis easily and diminish the need for further invasive diagnostic procedures.

## Introduction

Granuloma annulare (GA) is a self-limiting granulomatous disease of unknown etiology with a clear histopathological appearance. The GA lesions consist of typical palisading granulomas, with a central necrotic zone surrounded by radially oriented histiocytes, lymphocytes, and fibroblasts (1, 2). GA can present in a generalized fashion GA (GGA), localized (LGA), subcutaneous (SGA), and more rare variants of patchy and perforating GA.

GGA is defined as the presence of more than ten widespread skin lesions (3). A diverse assortment of therapies for GGA has been reported, yet missing an algorithm for treatment choices (4). LGA is characterized by less than ten asymptomatic skin papules with a circular appearance mainly localized on the dorsum of the hands and feet (5, 6). While LGA occurs in adults and children, SGA is a condition postulated to occur mainly during childhood (1, 2, 7). SGA consists of solid, non-tender, non-inflammatory, solitary, or multiple subcutaneous lumps. These lesions are commonly located above bony prominences, such as the anterior side of the lower legs, the dorsum of the hands and feet, and the scalp (5, 8). LGA and SGA lesions spontaneously regress without any treatment within a couple of years (3).

In most cases, diagnosing LGA and GGA as visible ring-shaped skin lesions is straightforward for a dermatologist. Unfortunately, this is rarely the case among pediatric patients with SGA lumps. Regardless of its harmless, self-resolving nature, SGA still is the most frequently biopsied, benign soft tissue mass in the lower extremity of children under the age of 5 (9, 10). The initial clinical impression about the sudden appearance of an unclear subcutaneous lump in a child can be either dubious or misleading, thus, making this disease notoriously difficult to diagnose (11–13). Although various imaging features of SGA lesions have been described in the literature, no specific hallmark is yet recognized to help clinicians distinguish SGA from other differential diagnostic considerations (9, 12–17). As a result, children often undergo several unavailing investigations before a biopsy is performed to rule out malignancy and establish an adequate SGA diagnosis. Incisional or excisional biopsy is the intervention preferred since punch biopsy may yield false negative results as it may miss the necrobiotic mucin-containing areas, characteristic of the SGA lesion (2, 5, 7).

We have performed this institutional review to investigate the current practice of diagnosing children with SGA, intending to find a specific imaging sign of this disease that can quickly and effectively achieve a definite, accurate diagnosis without needing a biopsy.

## Patients and methods

We retrospectively analyzed the electronic hospital records (medocs) of all children aged 0–18 years diagnosed with SGA from January 2001 to December 2020. Evaluation of the data according to the patient's disease history, management, and outcome is done. The information identified and analyzed for each patient included: sex, age at first presentation, the duration of symptoms and signs prior to the first presentation, the location of the lesion(s), the number of the lesions, associated pain, the clinical description of the lesion(s), the report of trauma prior to the appearance of the lesions, the time between the first presentation at our Department and treatment starting, the diagnostic imaging and lab work performed, the histopathologic report provided, the type of conservative or surgical treatment performed, the recurrence after treatment and the follow-up time from the first presentation. Three-dimensional reconstructions of MR images were performed with 3D Slicer (https://www.slicer.org/) version 4.7.0-2016-11-04 r25501 by manually segmenting the SGA lesions and superimposing them by volume renderings of the respective body regions in all relevant slices (18). After the procedure, volumes could be read out automatically with the “label statistics” module. The MR imaging studies were either performed on a Magnetom Sola or a Magnetom Symphony scanner with 1.5 Tesla magnetic field strength (Siemens Healthineers, Erlangen, Germany). Radiologists were confronted with different body regions, resulting in different examination protocols. Typically, the MRI protocol included two planes of T2-weighted fat-suppressed sequences and one T2-weighted sequence without fat-suppression. A T1-weighted sequence was also performed, typically in a coronal orientation. Diffusion-weighted imaging was invariably performed. Post-contrast T1-weighted sequences with fat-suppression were available in all situations. Descriptive statistical analysis was performed. This study has been approved by the Institutional Ethics Committee (EK-No. 33–126 ex 20/21), and informed consent was waived.

## Results

We identified 57 patients diagnosed with GA in the last 20 years. Twenty-nine of them were LGA cases and were excluded from the present study. Twenty-eight SGA patients were included for further analysis. Other variants of GA were not reported. The provided Table 1 illustrates in more detail the characteristics of every SGA patient included in our study.

**Table 1:** Characteristics of patients treated for subcutaneous granuloma annulare (SGA).

Patient	Age [y]	Sex	Number of Lesions	Lesion Location	Side	Diagnostics	Surgery	Recurrence	Other Diseases
1	2.5	M	1	Knee	R	US + x-ray + MRI	CE	No	–
2	2.5	F	1	Lower leg	R	US + x-ray	CE	Yes	–
3	4	M	1	Forearm	L	US + MRI	CE	No	–
4	4	F	1	Lower leg	L	US + x-ray + MRI	B	No	–
5	4.5	F	3	Head	R	US + MRI	B	No	–
6	3	F	1	Foot	L	US + MRI	CE	No	–
7	2.5	F	1	Lower leg	R	US + x-ray + MRI	CE	No	Atopic diseases
8	2.5	F	2	Hand and Thorax	L & R	US + MRI	CE	No	Juvenile Chronic Arthritis
9	4	F	1	Lower leg	L	US + x-ray + MRI	B	No	–
10	5	F	2	Lower legs	R & L	US + x-ray	–	No	–
11	2	M	2	Lower legs	R & L	US + x-ray	–	No	–
12	3.5	F	3	Lower legs	R & L	US + x-ray + MRI	B	No	–
13	4	F	2	Foot and Elbow	L & R	US	–	No	Atopic diseases
14	2.5	M	1	Foot	L	US	–	No	–
15	6	F	1	Lower leg	R	US + x-ray	–	No	–
16	4	M	1	Foot	L	US	CE	No	–
17	3	M	1	Pelvic crest	L	US	–	No	–
18	1	F	1	Lower leg	R	US + x-ray	–	No	–
19	12.5	F	1	Lower leg	R	US + x-ray	CE	No	Ulcerative Colitis
20	4.5	F	1	Lower leg	L	US + x-ray	–	No	–
21	5	M	2	Lower legs	R & L	US + x-ray	–	No	Urticaria
22	5.5	F	1	Hand	L	US	CE	Yes	
23	1	F	1	Lower leg	R	US + x-ray	–	No	
24	3.5	F	3	Lower legs & Foot	R & L	US + x-ray	B	No	–
25	2	F	2	Head	L	US + MRI	CE	No	–
26	5.5	F	1	Knee	R	US + x-ray	CE	No	–
27	5	F	1	Forearm	L	US + MRI	CE	No	–
28	3	M	2	Head	Frontal	US + MRI	B	No	–

28 patients diagnosed with SGA presented with a total of 41 lesions. Seven patients had two lesions each, and three patients had 3 lesions each. Abbreviations: M, male; F, female; R, right; L, left; MRI, magnetic resonance imaging; US, ultrasound; CE, complete excision; B, biopsy.

A strong female predilection of 2.5:1 is noted among our patients. The median age at the first presentation was 3.75 years (range 1–12.5 years). SGA lesions were mainly found on the lower extremities (*n* = 26). In 50% of the patients, the SGA lesions were located on the lower leg area, indicating the high clinical possibility of a typical location for an SGA lump. Ten patients presented with multiple lesions. The SGA lumps were described as solid, nontender, and without signs of inflammation. All children were otherwise healthy, and the SGA lumps were not associated with overlying cutaneous abnormalities.

The median time from the subcutaneous lump(s) appearance until the first presentation at the hospital reported by parents was 2 months (range 0.5–5 months). In 9 SGA patients (33%), parents reported that trauma preceded the appearance of the lump(s), and its persistence made them seek a doctor's opinion.

Every SGA patient included in the study has received an ultrasound examination (US) at the time of the first presentation. An unclear subcutaneous soft tissue mass with poorly defined borders, hypo- and hyperechoic zones, and mild vascularization was regularly described. In 16 SGA patients (57%), x-rays were performed at the time of the first presentation to exclude osseous abnormalities. Because of the several possible pathologies and the difficulty in excluding a potential malignancy, 12 patients (43%) were referred for magnetic resonance imaging (MRI), all of which had to be performed under general anesthesia. Yet, MRI findings did not yield a definitive diagnosis in any of the cases. The lesions were regularly described as subcutaneously located with ill-defined borders. Imaging led to the consideration of several differential diagnoses, most commonly a subcutaneous low-flow vascular anomaly, fibromatosis, fasciitis, and post-traumatic fat necrosis. Only in one case was SGA considered among the potential differential diagnoses.

Surgical intervention was performed in 18 patients (64%), including all who received an MRI. The median time between the first presentation of the child in the hospital and the surgical intervention was one month. Biopsy (*n* = 6) or complete excision (*n* = 12) was performed depending on the size of the lesion(s). Preoperative lab work included a complete blood count and basic metabolic panel, which resulted uneventfully in every case.

All patients who did not undergo surgery (10/28) were diagnosed by an attending specialist who recognized SGA after clinical and imaging examinations. These children experienced a spontaneous resolution of the subcutaneous lesion(s) which lasted from 7 to 29 months. Two of these patients received local corticosteroid treatment for 2 weeks, which led to a mild reduction of the lump(s) size, whereafter the local treatment was discontinued. The pediatric surgeons who clinically suspected these lesions to be SGA lesions decided on the clinical follow-up of these patients.

The median time of the follow-up was 19 months (range 14–48 months). Two patients who underwent complete surgical excision of the lump experienced a local recurrence occurring 4 and 5 months after surgery, respectively. Both recurrent cases resolved spontaneously 6 months later.

All patients were reviewed in November 2021 in the frame of the long-term follow-up. One girl had developed juvenile chronic arthritis 5 years after the biopsy for SGA, and another girl was diagnosed with chronic inflammatory bowel disease 9 years after having a complete excision of SGA.

**Figure 1:**
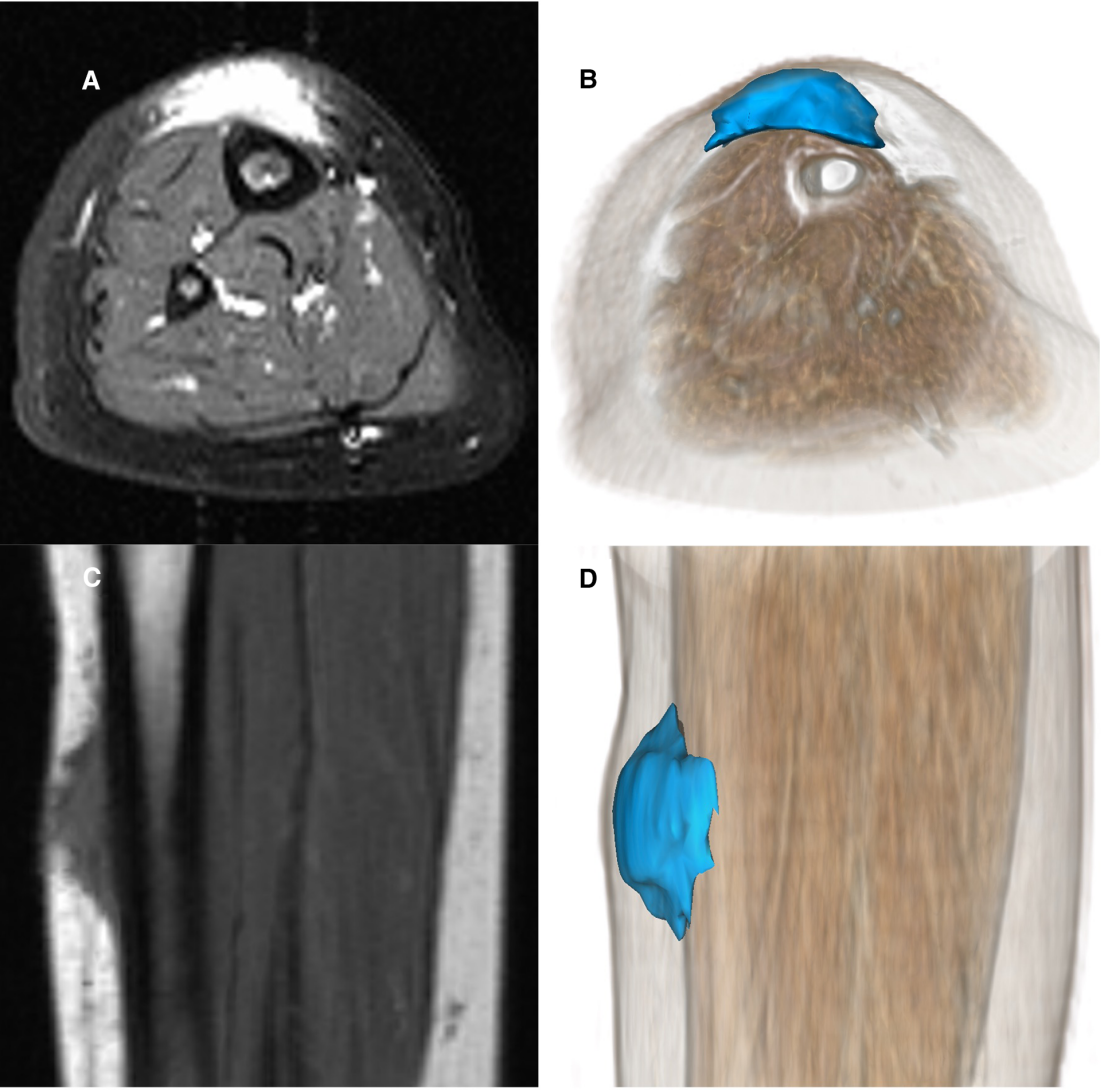
Representative MRI sections and 3D-reconstructions of subcutaneous granuloma annulare lesion (colored blue) on the right lower leg of a 2.5-year-old girl (**A–D**). A T2-weighted slice in axial orientation (**A**) shows the cap-shaped epifascial lesion with high signal intensity (bright). The corresponding 3D reconstruction is given in (**B**), visualizing the lesion in blue color and the remaining other tissues by overlaying a volume rendering in a skin-like color. (**C**) shows the lesion in a T1-weighted sagittal slice with low signal intensity (dark). The corresponding 3D rendering (**D**) demonstrates the cap-shaped morphology, extending from the deep fascia to the subcutaneous layer.

**Figure 2:**
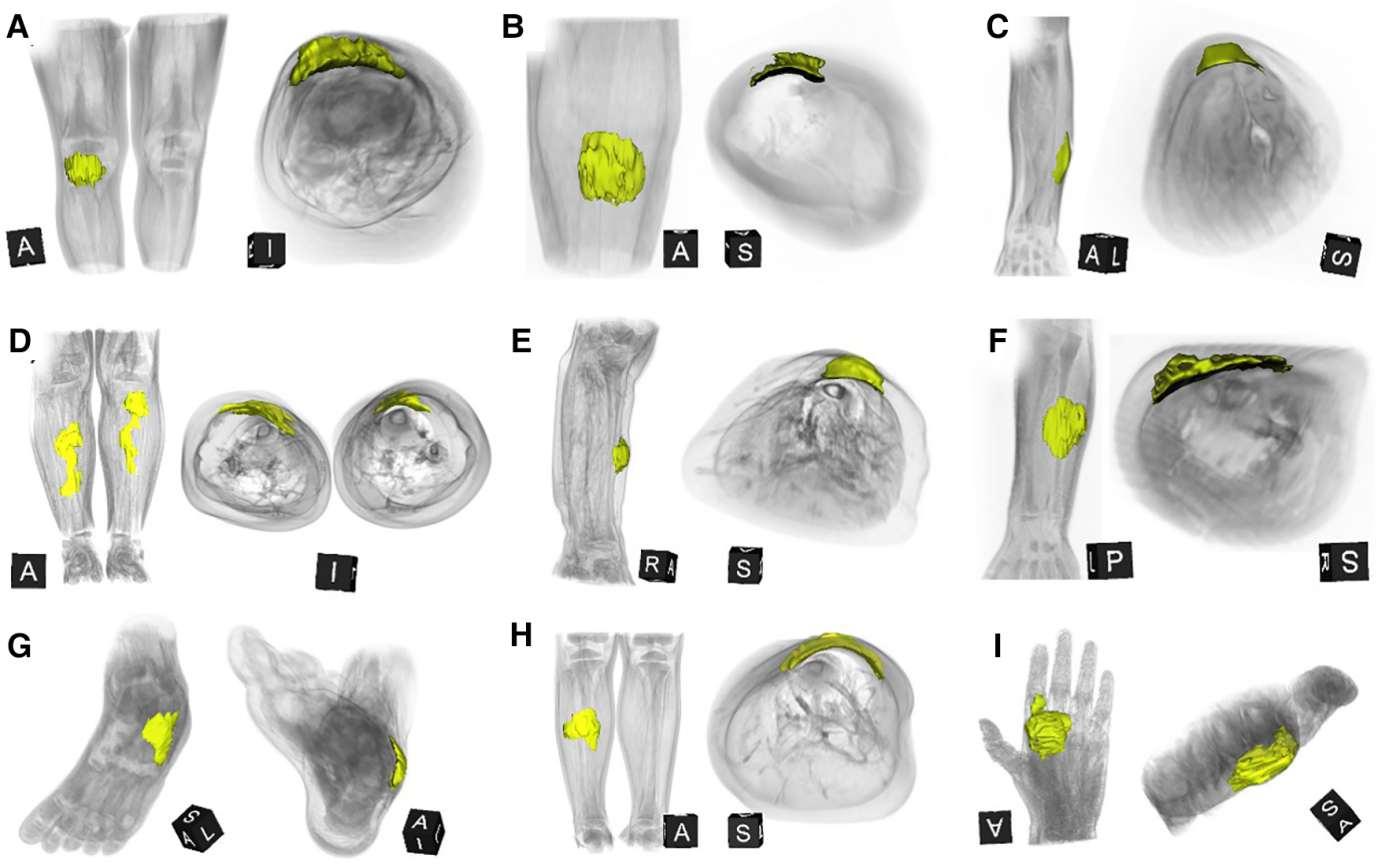
This composite figure shows 3D-reconstructions of magnetic resonance imaging (MRI) of all 9 patients with subcutaneous granuloma annulare (SGA) on the extremities who were evaluated by MRI. The 3D reconstructions of SGA (yellow) are overlaid by volume renderings of the respective extremity regions (gray) for reference. Note that all SGA lesions characteristically demonstrate a round or oval area with cap-shaped morphology, extending from the deep fascia into the subcutaneous layer. (**A**) right knee; (**B**) left lower leg; (**C**) left forearm; (**D**) right and left lower legs; (**E**) right lower leg; (**F**) left forearm; (**G**) left foot; (**H**) right lower leg; (**I**) left hand.

**Figure 3:**
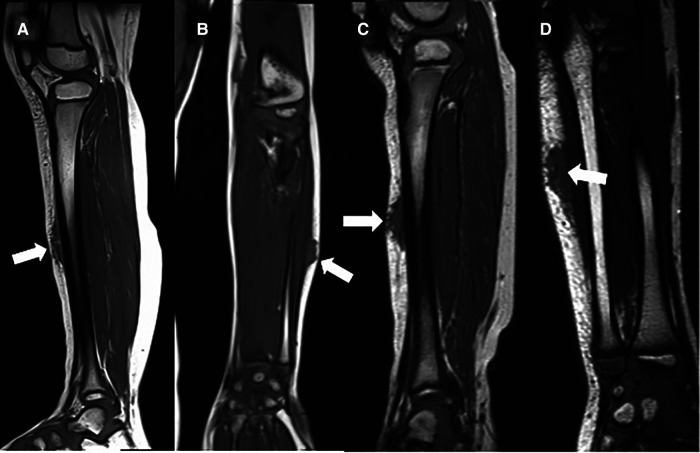
This composite figure shows representative T1-weighted magnetic resonance images (MRI) in sagittal or coronal orientation through the extremities of 4 children with subcutaneous granuloma annulare (SGA). Note the cap-shaped appearance of SGA lesions and that in T1-weighted MRIs they present as homogenous lesions (arrows) isointense relative to the muscles. (**A**) left lower leg, (**B**) left forearm, (**C**) right lower leg, (**D**) left forearm.

**Figure 4:**
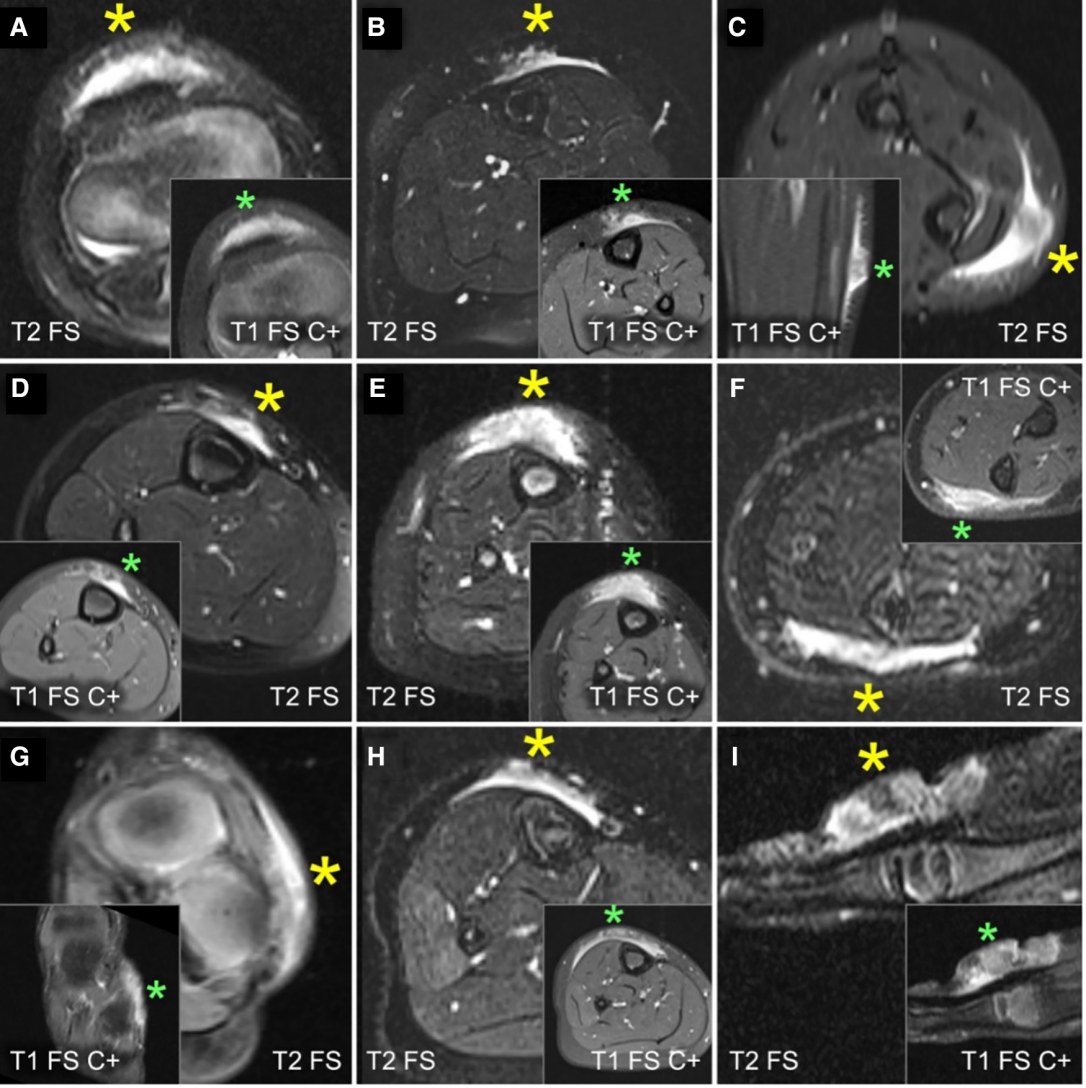
This composite figure shows representative slices of magnetic resonance imaging (MRI) through the subcutaneous granuloma annulare (SGA) on the extremities of 9 patients evaluated by MRI. The large boxes depict T2-weighted sequences with fat suppression (“T2 FS”). The small boxes depict T1-weighted sequences with fat suppression and intravenous contrast (“T1 FS C+”). Asterisks mark the locations of the SGA. A common finding in all presented cases is the cap-shaped lesion extending from the deep fascia into the subcutaneous fatty tissue. These lesions show heterogeneously hyperintense signal relative to the muscles in T2 FS images and marked contrast enhancement in T1 FS C+ . (**A**) right knee; (**B**) left lower leg; (**C**) left forearm; (**D**) right lower leg; (**E**) right lower leg; (**F**) left forearm; (**G**) left foot; (**H**) right lower leg; (**I**) left hand.

**Figure 5:**
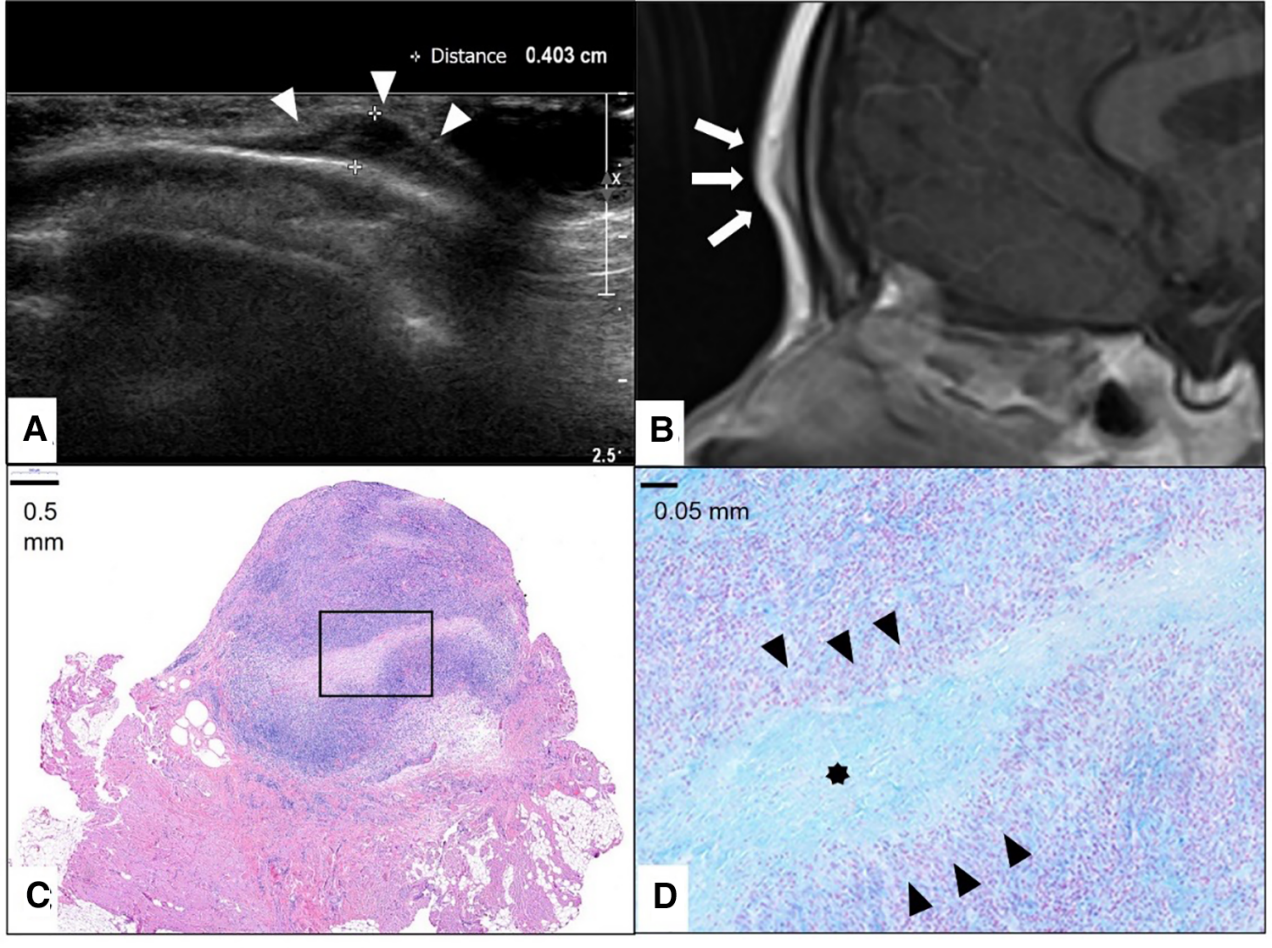
This composite figure shows a frontal location of the subcutaneous granuloma annulare (SGA). Note the epifascial cap sign in the ultrasound image marked with white arrowheads (**A**) and in the representative slice of magnetic resonance imaging (MRI) marked with white arrows. (**B**) The completely excised SGA lesion is localized in the subcutis overlying the fascia and is characterized by areas of necrobiotic granulomas shown here in hematoxylin and eosin staining. (**C**) The area marked with a box in (**C**) is enlarged in (**D**), showing alcian blue stain highlighting the mucin within the central zone of necrobiosis (star) surrounded by palisading histiocytes and lymphocytes (black arrowheads).

**Figure 6:**
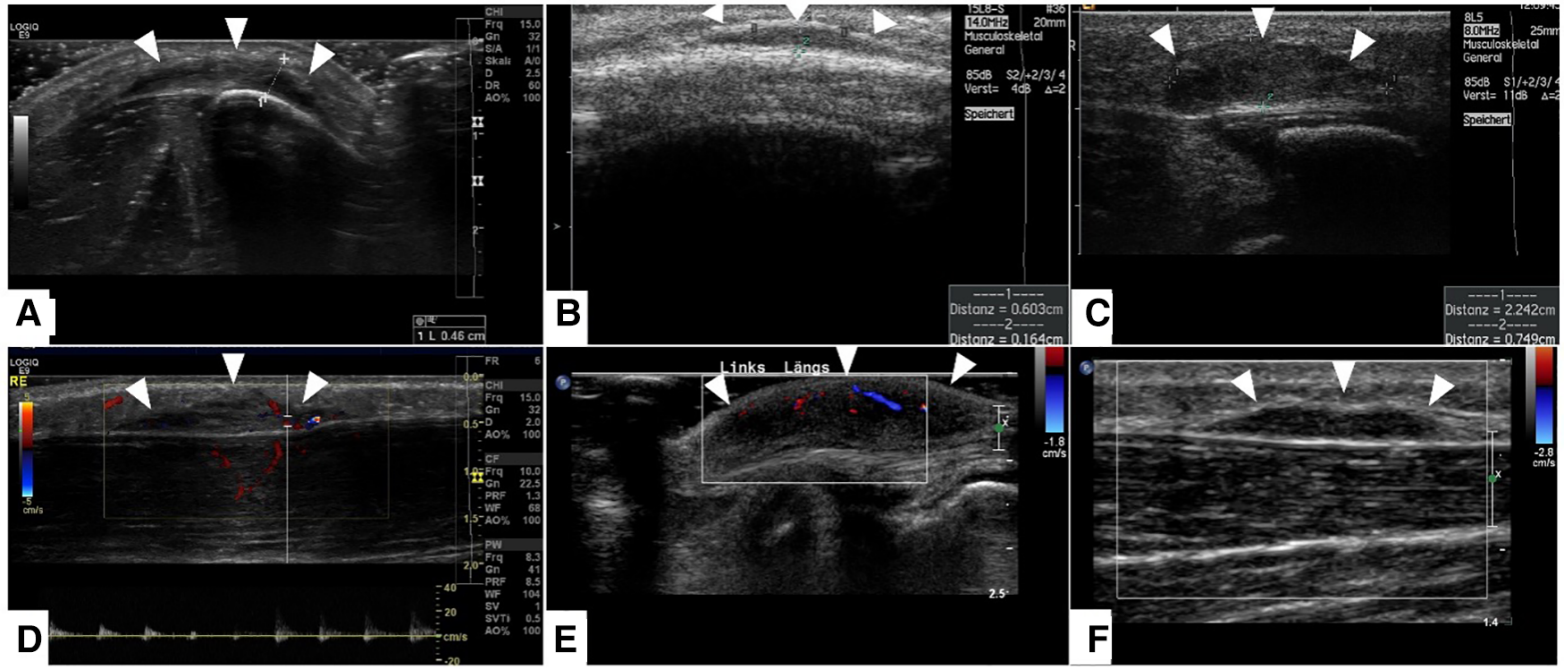
This composite figure shows representative slices of ultrasound imaging through the subcutaneous granuloma annulare (SGA) in 6 patients who had their final diagnosis by histopathology after excisional or incisional biopsy. A common finding in all presented cases is the cap-shaped SGA lesion extending from the deep fascia to the subcutaneous fatty tissue. The epifascial border is marked with white arrows. These lesions show heterogeneously hypoechoic signal in B-mode (**A–C**). The lesions stay hypoechoic and show mild perfusion in color mode US (**D–F**). (**A**)—right lower leg, (**B**)—scalp, (**C**)—right lower leg, (**D**)—right lower leg, (**E**)—left foot dorsum, (**F**)—left lower leg.

A careful retrospective review of all imaging led to the recognition of a novel and specific imaging sign for SGA, which we are not aware to be present in other pathologies. The SGA lesions show a well-defined broad-based rounded fascial border and an ill-defined crescent, cap-shaped epifascial border (Figure 1). We have named this typical shape of the SGA lesion “the epifascial cap sign”. The 3D reconstructions of 9 MR investigations in 9 children with SGA lesions on the extremities are performed to present the SGA lesions in a more illustrative way and are shown in Figure 2. They can help better estimate the lesion's location and shape, but regular MRI sequences are sufficient to identify the “epifascial cap sign”. The 3D analysis takes about 10 to 15 min for each lesion, mainly depending on the software solution available. SGA lumps mirror raised, rounded masses that project over the surface of the muscle's fascia without invading the underlying tissues. The SGA lesions are isointense relative to the muscles on T1-weighted images (Figures 1C, 3) and hyperintense relative to the muscles on T2-weighted images (Figures 1A, 4). In addition, the lesions are homogeneous in both T1 and T2 weighted MRI sequences and show variable enhancement after contrast material injection (Figure 4, small boxes). The MRIs of patients with SGA lesions on the head were performed to exclude intracranial and/or osseous abnormalities. The SGA lesions on the head had a median volume of 0.11 ml (range 0.05–0.14 ml) and were too small for a 3D reconstruction (Figure 5). The median volume of the SGA lesions on the extremities in patients receiving MRI was 2.7 ml (range 1.3–6.3 ml). A detailed retrospective review of the archived ultrasound images of our SGA patients confirmed the cap shape of SGA lesions (Figure 6).

**Figure 7:**
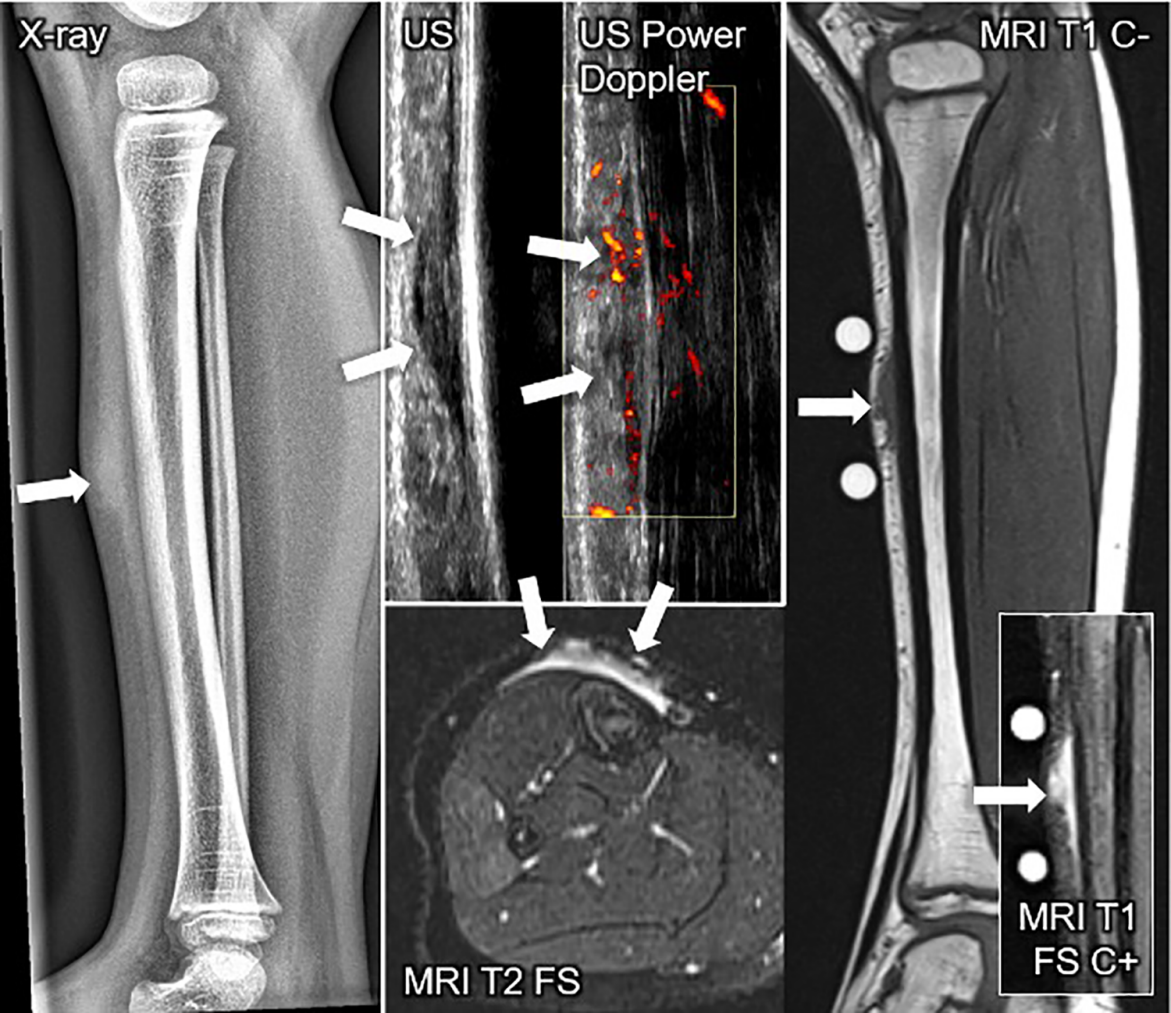
This composite figure shows various imaging methods used to evaluate a firm, immobile, indolent subcutaneous lesion in the mid-pretibial region of the right lower leg of a 4-years old girl. The lateral x-ray view of the lower leg depicts a thickened pretibial subcutaneous tissue (arrow), but no osseous abnormalities. The ultrasound image (US) shows a hypoechoic lesion in the typical epifascial cap shape (2 arrows) characteristic of subcutaneous granuloma annulare (SGA), confirmed by histopathological examination. Power Doppler US indicates slight hyperperfusion (2 arrows) in the area of SGA as compared to the surrounding tissue. In T1-weighted magnetic resonance images (MRI T1 C-), SGA presents as a homogenous lesion (1 arrow) isointense relative to the muscles. In T2-weighted images with fat suppression (MRI T2 FS), SGA shows a heterogeneously hyperintense signal relative to the muscles (2 arrows) extending from the muscular fascia into the adjacent subcutaneous tissue in a cap shape. Finally, in T1-weighted images with fat suppression and intravenous contrast (MRI T1 C+) SGA shows a marked contrast enhancement (1 arrow).

## Discussion

In the present study, we show for the first time that SGA lesions mirror a raised-rounded epifascial cap with a broad circular base and a continuous curved surface shape that extends towards the more superficial tissues. This shape of SGA is characteristic of this disease and, to the best of our knowledge, is not found in any other subcutaneous lesions.

Most SGA cases occurred around the age of 4 years (Figure 7) with greater frequency in girls, confirming the female predilection of this disease, as reported in previous studies (14, 19–21). Our investigation highlights that children with SGA present with painless, nonmobile, subcutaneous lumps on their lower extremities, mainly on the pretibial area and other trauma-exposed bony prominences, like the scalp, the ulnar side of the forearms, hands, and feet. Children are otherwise healthy, and the SGA lumps are not associated with overlying cutaneous abnormalities.

Trauma has repeatedly been reported as one of the triggering events in the etiology of SGA (22). However, the pathophysiologic mechanisms behind it are still unknown. Local trauma has also been reported in one-third of our patients. Therefore, it is possible that lesions on the lower extremities, especially areas vulnerable to trauma like the pretibial areas, are related to repetitive, sustained injuries from minor events during physical activities. Minor trauma of the bony prominences in the pediatric age group is a normal and common event, elucidating further the predisposition of the pediatric population to this condition. However, in most cases, a history of injury cannot be obtained, perhaps due to the prolonged interval between trauma and the initial observation of the lesion or other unknown factors. Thus, there must be other overriding factors, the presence or absence of which determines whether or not children develop SGA.

In the absence of a trauma history, the clinical picture usually resembles a manifestation of a neoplastic disorder in the eyes of a physician unfamiliar with the SGA entity. As a matter of fact, despite the harmless nature of the SGA lumps, a large portion of our patients underwent either incisional or excisional biopsy of the painless lump due to an unclear diagnosis and fear of a possible malignant trait. Indeed, according to a review from Chung et al., SGA is the most frequently biopsied, benign soft tissue mass in children under 5 despite its very low clinical incidence in the United States (9).

Few studies have described the MRI characteristics of the SGA lesions but failed to find a common pathognomonic imaging pattern, enabling clinicians to properly diagnose and differentiate SGA from other possible diseases based solely on imaging characteristics (9, 11, 14, 16, 23). Our present study recognized a reproducible shape of the SGA lesions, consistent in MR imaging of all children with SGA. This typical shape of SGA nodules mirrors a raised-rounded cap projecting on the surface of the muscle's fascia, which can be reliably used as a typical imaging shape for SGA lumps in MRI. Recognition of this sign in an otherwise healthy child presenting with a symptomless subcutaneous swelling should lead clinicians to suspect SGA, recommend a follow-up visit in 4 weeks, and prevent the majority of children with SGA from undergoing further invasive diagnostic procedures. We believe this sign to be the typical sign for SGA as, to the best of our knowledge, we are unaware of another subcutaneous disease that presents in this shape. Because SGA is a self-limiting disease, we speculate that the tissue characteristics of SGA lesions might change over their lifetime. In addition, the number and size of necrobiotic areas might also influence the imaging appearance of these lesions. However, the epifascial cap shape of the lesions seems not to be significantly impacted. Experience with SGA in terms of characteristic imaging findings is limited. Lack of awareness is believed to be the most crucial factor in why differential diagnoses did not include SGA, as in the present study. Learning new patterns or finding more specific signs in rare lesions like SGA is challenging.

The epifascial cap shape of the SGA lesions is also evident in US examinations, even though our retrospective analysis of the US images has limited value and reliability due to inter-examiner differences and the lack of standardized guidelines for image assessment of subcutaneous tissues. The US has three major obstacles which are difficult to standardize: the cooperation of a young patient, the subjectivity of the examiner, and the intensity of pressure applied to the lesions with the US probe, which often alters the shape of the subcutaneous lesions. On the US images of most of our SGA patients, we have detected the same cap-shaped presentation located on the muscle's fascia compatible with the sign as it is seen on the MRI images of these patients. US findings were otherwise described as solid nodules with ill-defined borders and noted central hypoechoic zone surrounded by a hyperechogenic periphery, agreeing with the sonographic characteristics of SGA reported in a few previously published studies (11, 12, 15, 17, 24–26). MRI is often performed because it is one of the most specific imaging modalities to noninvasively clarify lesions whose entities have remained unclear. However, MRI is expensive, and downside of MRI is that in children under the age of 7, it often requires general anesthesia. It would be an outstanding achievement to diagnose SGA based on US findings alone. However, it must be considered that the incidence of SGA is low, impairing the potential gain knowledge at a high rate.

In addition, we have reviewed all previously published cases of SGA that have provided MR or US imaging of the lumps and confirmed that every reported image in the literature so far consistently presents as an epifascial cap formation (9, 11, 12, 14–16, 19, 20, 24–30). The only reference in the current literature we found to describe different MR characteristics of the SGA lump as compared to what we have just described concerns an unusual infiltrative forearm lesion in a 3-year-old girl with juvenile-onset diabetes (31). According to the authors of this report the lesion disappeared one month after the biopsy and when the glycemic control was achieved.

There are numerous subcutaneous pathologies in children, with both specific and unspecific imaging findings. In some diseases, subcutaneous lesions can be one of several clinical signs in the context of systemic diseases such as rheumatoid arthritis, sarcoidosis, tuberculosis, and others, with imaging findings that are not specific. Most of these lesions present as nodules, which can be singular or multiple, and vary in size. The nodules are mobile, show irregular shape and margins in imaging, and enhance after the application of a contrast agent in MRI, due to inflammation [10, 14, 15]. Therefore, clinical correlation and, if necessary, histological confirmation of diagnosis are crucial for accurate diagnosis and management of subcutaneous pathologies that present in the nodular form. To the best of our knowledge, these lesions have not been reported in the literature as presenting in the “epifascial cap” shape, as observed in completely healthy children with SGA. Given the typical cap shape of each SGA lesion, the initial still illusive stimulus that triggers the recruitment of the inflammatory particles may lie in the subcutaneous tissue. These inflammatory cells migrate along the fascia and accumulate to form a tight aggregate around the inflammatory stimulus. The activated inflammatory cells seem unable to efficiently remove the inflammatory stimulus, leading to a secondary inflammatory response that ultimately gives rise to granuloma formation. SGA granulomas consist of central necrosis surrounded by histiocytes, eosinophils, and lymphocytes in a tier-like fashion. This massive tight aggregate of inflammatory cells around the stimulus probably explains why SGA often presents as a non-vascularized immobile subcutaneous lesion, even though peripheral vascularization has often been described in color Doppler mode (24, 25). Elucidating the pathogenesis of SGA seems challenging, but our understanding is constantly evolving. Recently a study by Min et al. reported the upregulation of Th1 and Th2 pathways in these lesions, and a 2021 study by Wang et al. found activation of the Th1 and JAK-STAT pathways in their cohort (32, 33).

The hypothesis of granuloma formation on an autoimmune basis and its association with diabetes mellitus, rheumatoid disease, and other systemic disorders have been investigated in several studies (34–36). A study by Grogg et al. (21) reported 2 cases of diabetes mellitus in 34 SGA patients. Yet, Felner et al. (34) found no significant correlation between diabetes mellitus and SGA in their cohort of 47. None of the children in our study developed diabetes mellitus during the long-term follow-up. Even though further evidence is needed to support any possible association, we have noted that occasional SGA female patients seem susceptible to developing autoimmune conditions later in life, as one case of juvenile chronic arthritis and one chronic inflammatory bowel disease were also reported in our cohort. However, it is unclear if this occasional association is related to the clear female gender bias of autoimmune disorders in general, as SGA also occurs more frequently in females. The association of SGA lesions with vascular and connective tissue diseases is also often discussed (21, 37), but none of the children in our study presented with vascular changes or connective tissue disorders. While many inconsistencies exist in the published literature, further research is needed to clarify the discrepancies and draw definite causal conclusions about this rare disease.

The epifascial cap is a typical imaging sign of SGA lesions which, to the best of our knowledge, distinguishes this disease from other subcutaneous lesions. Recognition of this sign in an otherwise healthy child presenting with a symptomless subcutaneous lump over bony prominences should enable clinicians to diagnose SGA accurately, schedule the patient for a follow-up visit and prevent the majority of children with this disease from undergoing further invasive diagnostic procedures.

## Data Availability

The raw data supporting the conclusions of this article will be made available by the authors, without undue reservation.
